# Comparison of removed dentin thickness with hand and rotary instruments

**Published:** 2009-04-17

**Authors:** Shahriar Shahriari, Hasan Abedi, Mahdi Hashemi, Seyed Mohsen Jalalzadeh

**Affiliations:** 1 *Department of Endodontics, Dental School, Hamadan University of Medical Sciences, Hamadan, Iran.*; 2 *General practitioner, Hamadan, Iran*.

**Keywords:** Instrumentation, ProFile, Root canal preparation, Stainless Steel, Technique

## Abstract

**INTRODUCTION:** The aim of this study was to evaluate the amount of dentine removed after canal preparation using stainless steel (SS) hand instruments or rotary ProFile instruments.

**MATERIALS AND METHODS:** Thirty-six extracted human teeth with root canal curvatures less than 30º were embedded in clear polyester resin. The roots were cut horizontally at apical 2, 4 and 7 mm. Dentin thickness was measured at each section and the sections were accurately reassembled using a muffle. Root canals were randomly prepared by SS hand instruments or rotary ProFile instruments. Root sections were again separated, and the remaining dentin thickness was measured. Mann-Whitney *U* and t tests were performed for analytic comparison of the results.

**RESULTS:** The thickness of removed dentin was significantly different between the two used methods (P<0.05). Significantly greater amounts of dentin was removed mesially in all sections in hand instrumentation group (P<0.001).

**CONCLUSION: **ProFile rotary instrumentation prepares root canals with a greater conservation of tooth structure.

## INTRODUCTION

The quality guidelines of the European Society of Endodontics state that elimination of residual pulp tissue, removal of debris and maintenance of the original canal curvature during enlargement are the main objectives of root canal instrumentation (1). An ideal prepared root canal should have a progressively tapering conical shape which preserves the apical foramen and the original canal curvature without transportation (2). Several studies have concluded that none of the instrumentation techniques or devices currently used can completely clean root canals, especially curved roots (3-5). Arguably the cleaning ability of manual root canal instrumentation has been shown to be superior to that of automated devices (3,6). However, it was recently demonstrated that instrumentation with automated devices using rotary nickel-titanium (Ni-Ti) instruments with various tapers led to promising results; *i.e.* less straightening or decentralization of the canal, and a rounder canal preparation even in severely curved root canals (7-11). The thickness of the remaining dentine following intraradicular procedures may be the most important iatrogenic factor that correlates to incoming fracture resistance of the root (12). Excessive flare of coronal third is not necessary, since flaring could considerably reduce the residual dentin leading to greater susceptibility to vertical root fractures (13). Moreover, preparation of the apical third can also reduce residual dentin resulting in weakened apical root structure. This is particularly important in a root with an oval cross section (*e.g.* mesial roots of mandibular molars) (14,15).

On the other hand, there are other studies that report dentin removal may not necessarily increase the risk of fracture (16) and that there was no significant difference in fracture load between teeth prepared by SS hand instruments or those prepared by Ni-Ti rotary instruments (17).

Lim and stock suggested 0.3 mm of dentin as a minimum thickness of canal walls that should remain after canal preparation. This allows adequate resistance against lateral forces during canal obturation and occlusal forces (18). Caputo and Standlee reported that 1 mm of tooth structure around the post is required for resistance against vertical fracture (19). Katz and Tamse confirmed that in an oval root-canal space, mesial and distal sides of root canal are mainly affected by intraradicular procedures. The majority of dentine removal occurs during the root canal preparation, and less structure is lost during post space preparation, with most losses in the mesial and distal directions (20).

The comparative evaluation of dentin removal efficacy between ProFile rotary system and SS hand instrumentation has not been studied. Therefore we ventured to compare the removed dentin thickness in mesiobuccal (MB) canals of mandibular molars at 2, 4 and 7 mm from the anatomic apex, after canal preparation with stainless steel (SS) hand instruments and rotary ProFile instruments.

## MATERIALS AND METHODS

Thirty-six untreated human mandibular molars with mature apices and intact roots were used in this experimental study. Teeth were stored in 10% buffered formalin solution. An endodontic access cavity was prepared, and the distal roots were resected. A patency K-file size #10 (Dentsply, Maillefer, Switzerland) was passively introduced into the mesiobuccal canals until it became visible from apical foramen. Working length was established at 0.5 mm short of this point (21). Radiographs were taken with E-speed film from the buccal and mesial aspects, (Kodak Co., New York, USA) in order to determine canal curvature. The angle of the curvature was measured according to the Schneider’s method (22). Canals with a curvature greater than 30˚ were excluded.

A simple and accurate modified Bramante Muffle system was used in this study (23,24). The teeth were embedded in clear polyester resin using small cylindrical investment molds. The tooth crown was protruded to the level of CEJ and the root was placed parallel to the long axis of the mold and stabilized with blue dental wax. Clear polyester resin was poured circumferentially until the resin was flushed with the root tip. After the resin was completely set, they were removed from the mold. Three holes with 0.3 mm diameter and 120˚ apart were drilled parallel to the long axis of the block for placement of a bolt at a later stage. A 2 mm-deep orientation groove was prepared with a low-speed round bur (ISO #21; Dentsply Maillefer, Ballaigues, Switzerland) along the surface of the resin block facing the MB canal. The embedded roots were cut horizontally at 2, 4 and 7 mm short of the anatomic apex with a 0.3mm-thick blade mounted on a precision saw (25). Dentin thickness was measured at each section from mesial, distal, buccal and lingual directions using a calibrated stereomicroscope (Olympus, Tokyo, Japan) at ×4 magnifications with 0.01 mm accuracy.

The sections were then reassembled according to the orientation grooves. Three 0.3-mm bolts were inserted in the predrilled holes and secured by matching nuts. All root-canal preparations and dentin thickness measurements were performed by the same operator before and after instrumentation.

The teeth were randomly assigned with the aid of a computer algorithm (26) to create 2 groups of eighteen each; the mesiobuccal canals in each group were prepared as follows:

1) *SS hand instruments: *root canals were prepared using the step-back technique with SS hand files (Dentsply, Maillefer, Switzerland). The apical part was widened by filing and watch-winding movements using file size #35 as the master apical file. The canal was successively flared using files #40, 45 and 50. Finally, middle and coronal portions of the canal were prepared and shaped using Gates Glidden drills #2 and 3 (Mani Inc., Japan). The root canals were frequently irrigated with 2 mL normal saline.

2)* Rotary profile instruments: *samples were prepared by crown-down technique using Ni-Ti ProFile rotary files. These instruments were set into constant rotation with a 128:1 reduction handpiece (Anthogyr, Sallanches, France) powered by an air motor according to the manufacturer’s instruction.

The rotary files were placed with a gentle picking motion for 10 second. The preparation sequence was as follows 0.06 taper-size 25, 0.06 taper-size 20, 0.04 taper-size 30, 0.04 taper-size 25 and 0.04 taper-size 20. Apical preparation to the working length was performed using 0.04 taper size 35. The canals were continually irrigated with 2 mL normal saline using a plastic syringe.

**Table 1:** Mean of removed dentin thickness after instrumentation^a^

**Distance from apex**	**Ni-Ti Rotary instrumentation**			**SS Hand instrumentation**		
	**B** ^b^	**M** ^c^	**D** ^d^	**B**	**M**	**D**
**2(mm)**	0.127±0.174	0.048±0.281^e^	0.136±0.075	0.275±0.199	0.341±0.229^e^	0.146± 0.235
**4(mm)**	0.299±0.425	0.148±0.089^e^	0.186±0.164	0.482±0.294	0.415±0.307^e^	0.290±0.224
**7(mm)**	0.341±0.259^e^	0.246±0.255	0.183±0.176^e^	0.586±0.318^e^	0.311±0.273	0.494±0.274^e^

*a.* *mean±SD in mm b. Buccal Dimension, ****c.**** Mesial Dimension, ****d.**** Distal Dimension, ****e. ****Differences is significant (P<0.05)*

Sections were separated, and the individual dentin thicknesses determined. The average measurement at each root section was calculated and recorded. The removed dentin thickness was calculated as the difference in mm measured before and after instrumentation. Since the values of dentin thickness in the lingual dimension included the diameter of the mesiolingual canal and isthmuses that were not instrumented, they were excluded from the results.

The data was analyzed with SPSS version 15 using Kolmogrov-Smirnov test for normality, Leven’s Test for equality of variances and t-test with α<0.05 as the level for statistical significance.

## RESULTS

The amount of dentin removed at buccal, mesial, and distal aspects are presented in Table 1. These numerical values show that hand instruments removed more dentin than rotary ProFiles in all sections. Statistical analysis found significant differences between the two techniques at 2 and 4 mm sections in mesial direction (P=0.002), and 7 mm in distal and buccal directions (P<0.001 and P=0.016 respectively).

The residual dentin thickness after instrumentation is presented in Table 2. Differences between rotary and hand instrumentation was significant at the 7 mm section in distal direction (P<0.05).

## DISCUSSION

Bio-mechanical preparation of the root canals is one of the most important stages of a root canal treatment. Original canal curvature, especially at the apex and inner side of the root curvature should be preserved during canal shaping. In this regard, any straightening which might interfere with canal integrity has to be prevented (2,10,27).

This study revealed that in all dimensions SS hand instrumentation removed more dentin than Ni-Ti rotary ProFile instrumentation. This finding is consistent with other studies that found Ni-Ti rotary files able to prepare curved root canals with greater conservation of tooth structure (11,28).

It should be noted that the findings of this study are restricted by variables such as age and properties of dentin. While severely curved canals were excluded, the various degrees of root curvature would also clearly affect the results. Moreover, it may be more appropriate to conduct such measurements using more accurate techniques such as the µCT and with a greater number of cross-sections.

In this study, dentin thickness was measured before and after instrumentation. These values suggested that the mechanical limits of the instruments used to enlarge the root canal to approximate predetermined values would significantly weaken the dentinal walls. It has been indicated that 0.3 mm of canal wall should exist after canal preparation as the minimum remaining dentin thickness (RDT). This is important for providing enough resistance against lateral forces during canal filling and occlusal forces (18). On the other hand, Sathorn *et al.* (16) suggested that removal of dentin may not necessarily lead to an increased risk of tooth fracture, and that a multitude of variables exist. In this study an RTD less 0.3 was observed in seven and four samples with SS hand and rotary instruments respectively. This was observed at 2 mm sections.

It has been shown that at the mid-root, the SS files caused more enlargements toward the inner part (danger zone), but at the apical level, these files caused more enlargement toward the outer part compared to the Ni-Ti files (10). This demonstrates that Ni-Ti rotary instruments decrease the risk of strip perforations in danger zones and straightening in curved root canals.

**Table 2:** Mean of residual dentin thickness after instrumentation

**Distance from apex**	**Ni-Ti Rotary instrumentation**			**SS Hand instrumentation**		
	**B** ^a^	**M** ^b^	**D** ^c^	**B**	**M**	**D**
**2(mm)**	0.8706±0.324	0.6894±0.317	0.5117±0.187	0.8706±0.365	0.5500±0.285	0.4417±0.155
**4(mm)**	1.0328±0.395	0.7539±0.190	0.7272±0.232	1.1328±0.421	0.7144±0.304	0.6528±0.237
**7(mm)**	1.2456±0.378^d^	0.9722±0.219	0.9339±0.143	1.2650±0.443	0.8694±0.259	0.6328±0.244^d^

*a.* *Buccal Dimension,* ***b.*** *Mesial Dimension,* ***c.*** *Distal Dimension,* ***d.*** *Differences is significant (P<0.05)*

The removed dentin thickness in the distal dimension of 7-mm section (inner side of the root curvature in the mid-root) and in the mesial dimension of 2-mm and 4-mm sections (outer side of the root curvatures in the apical zone) by the SS hand instruments were significantly higher compared to those by the rotary ProFile instruments in the current study. This might be due to the shape memory of SS hand instruments. When SS instruments are placed in curved root canals they will tend to reverse back to their straight shape leading to excessive removal of dentin in the outer surface of curvature at the apical zone, as well as the inner surface of the curvature at mid-root.

Excessive removal of dentin in the 7-mm section (danger zone) may lead to strip perforation. During canal preparations, particularly when using SS hand instruments, attention should be paid to the danger zones and the amount of dentin removed. The use of anti-curvature technique has an important role in preparation of the curved root canals (27).

ProFile rotary instrumentation can prepare root canals with greater conservation of tooth structure. Further research particularly clinical trials, are required to confirm the advantages of rotary instruments with regard to the tissue conservative.

## CONCLUSION

Within limitations of this study, SS hand instruments removed more dentin in all dimensions than Ni-Ti rotary ProFile instruments. Significant changes were found in the remaining dentin thickness between two groups in mesial direction at the apical zone, and in distal and buccal directions in the mid-root.
